# The role of (pro)renin receptor and its soluble form in cardiovascular diseases

**DOI:** 10.3389/fcvm.2023.1086603

**Published:** 2023-02-02

**Authors:** Boyang Wang, Haipeng Jie, Shuangxi Wang, Bo Dong, Yunzeng Zou

**Affiliations:** ^1^Department of Cardiology, Shandong Provincial Hospital, Cheeloo College of Medicine, Shandong University, Jinan, China; ^2^Department of Cardiology, Shandong Provincial Hospital Affiliated to Shandong First Medical University, Jinan, China; ^3^Department of Cardiology, Shandong University of Traditional Chinese Medicine, Jinan, China; ^4^Key Laboratory of Cardiovascular Remodeling and Function Research, Qilu Hospital, Shandong University, Jinan, China; ^5^Shanghai Institute of Cardiovascular Diseases, Zhongshan Hospital and Institutes of Biomedical Sciences, Fudan University, Shanghai, China

**Keywords:** hypertension, fibrosis, cardiovascular disease, renin-angiotensin system, (pro)renin receptor

## Abstract

The renin-angiotensin system (RAS) is a major classic therapeutic target for cardiovascular diseases. In addition to the circulating RAS, local tissue RAS has been identified in various tissues and plays roles in tissue inflammation and tissue fibrosis. (Pro)renin receptor (PRR) was identified as a new member of RAS in 2002. Studies have demonstrated the effects of PRR and its soluble form in local tissue RAS. Moreover, as an important part of vacuolar H^+^-ATPase, it also contributes to normal lysosome function and cell survival. Evidently, PRR participates in the pathogenesis of cardiovascular diseases and may be a potential therapeutic target of cardiovascular diseases. This review focuses on the effects of PRR and its soluble form on the physiological state, hypertension, myocardial ischemia reperfusion injury, heart failure, metabolic cardiomyopathy, and atherosclerosis. We aimed to investigate the possibilities and challenges of PRR and its soluble form as a new therapeutic target in cardiovascular diseases.

## 1. Introduction

As a classic and pivotal target for the treatment of cardiovascular diseases (CVD), the renin-angiotensin system (RAS) has received sustained and extensive attention. The importance of circulating RAS in the regulation of fluid homeostasis and cardiovascular disease has been well acknowledged. Circulating RAS principally contains two axes. The classic axis is composed of renin, angiotensinogen (AGT), angiotensin (Ang) I, angiotensin-I-converting enzyme (ACE), and Ang II. The non-classical RAS axis, which mainly consists of ACE2, Ang 1–7, and Mas receptor (MasR), antagonizes the classical axis and has a protective role (1). The imbalance of the two axes is an important factor in the occurrence and development of CVD caused by circulating RAS (1). In recent years, local RAS that appears in tissue has also aroused widespread concern in studies; it is widely involved in sympathetic outflow, tissue inflammation, oxidative stress, tissue fibrosis and a series of pathological processes, promoting CVD progression in conjunction with circulating RAS (2). (Pro)renin receptor (PRR) was identified as a new member of the local RAS by Nguyen et al. in 2002 (3). During the past decade, an increasing number of studies have revealed that PRR is involved in cardiovascular disease progression as part of the local RAS (4). In our research, we further found that PRR participates in the pathogenesis of diabetic cardiomyopathy (DCM) (5), alcoholic cardiomyopathy (6), and aneurysm (7).

Cardiovascular disease is a common health issue and has been a major limiting lifetime factor. As one of the leading causes of death globally, it has contributed to nearly 40% of deaths in the aging population (8). Mortality has shown a declining trend in recent years. Nevertheless, the incidence of CVD is increasing year by year, especially in high-income countries. Age is the main risk factor for CVD; worse, the world’s elderly population is experiencing an unprecedented increase, and by 2030, it is estimated that the elderly population will reach approximately 20% of the total population; in China, this number is forecasted to reach 30% (9), which places a huge burden on the social economy. In recent years, significant progress has been made in the management of cardiovascular diseases; however, patients with CVD still have high mortality rates and low quality of life. Hence, there is an urgent need to search for new therapeutic targets and establish more effective treatment strategies.

In this review, we mainly summarized the role and controversies of PRR and its cleaved product soluble PRR in cardiovascular pathogenesis and their prospective guiding roles in further research and clinical application.

## 2. Biochemical characteristics of PRR and sPRR

### 2.1. PRR

(Pro)renin receptor, also known as ATP6AP2, is a single-pass transmembrane protein composed of 350 amino acids. It consists of an N-terminal extracellular domain composed of a hydrophobic region (amino acids 1-16), a transmembrane domain and a short cytoplasmic domain (3, 10). The gene that codes PRR is located on the X chromosome (Xp11.4). Full-length PRR expression was found to be higher in thyroid, brain, kidney, adrenal, endometrium, heart, appendix and 18 other tissues and lower in pancreas and salivary gland (11), and it is mainly present on endomembrane systems, including vacuolar membranes and plasma membranes (10).

**FIGURE 1:**
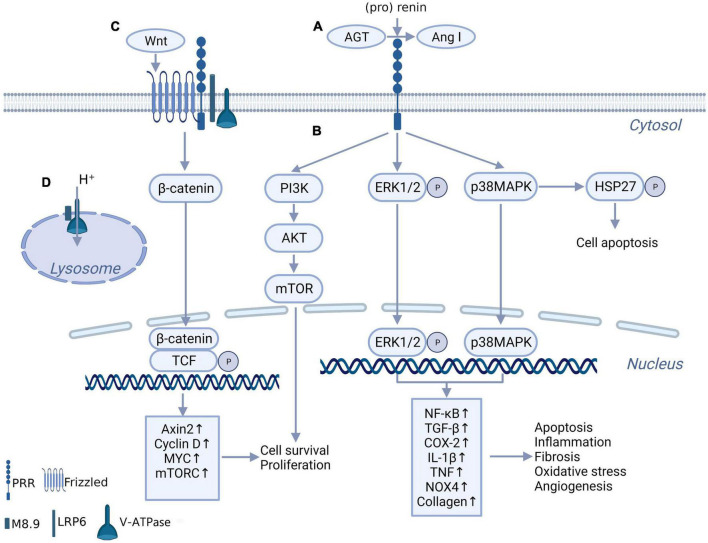
The biochemical function of PRR. **(A)** PRR convert AGT to Ang I through binding with (pro) renin, independent of renin. **(B)** When bind with renin and (pro) renin, PRR promotes multiple intracellular pathways, include ERK1/ERK2 pathway, p38MAPKs-HSP 27 pathway and PI3K-AKT pathway, and further promotes downstream transcription factors expression. **(C)** PRR constitutes Wnt receptor complex together with Frizzled, H + -ATPase and LRP6, takes part in Wnt/β-catenin pathway. **(D)** The truncated form of PRR named M8.9 is an important part of H^+^-ATPase, contributes to normal lysosomal function.

As PRR was first reported, it was found to facilitate AGT cleavage and increase angiotensin (Ang) II production and function (3, 12). The binding of (pro)renin to the PRR triggers a conformational change and non-proteolytic activation of (pro)renin, resulting in Ang I being derived from AGT (Figure 1A). ACE further converts Ang I to Ang II. This discovery may explain the issue of how the low level of renin maintains a high level of Ang II activity in the brain (13) and make PRR a new member of the RAS system. It was clear that the effect of PRR enhancing the generation and action of Ang II in the brain plays a role in neurogenic hypertension (4, 14). However, compared with the Ang II-dependent pathway of PRR, the intracellular signaling molecules activated by PRR independent of Ang II might be more important in inflammation and fibrosis of the myocardium, kidney, and other tissues in pathologic conditions and have received more attention. When binding with (pro)renin, PRR directly activates the downstream intracellular signaling pathways, including the extracellular signal-related protein kinase (ERK) 1/ERK2 pathway, p38 mitogen-activated protein kinases (p38MAPKs)–heat shock protein (HSP) 27 pathway and phosphatidylinositol 3-kinase–p85α (PI3K-p85α) pathway, independent of Ang II (15–17) (Figure 1B). This process triggers a sequence of cascade reactions and ultimately upregulates a series of nuclear factors, which are important contributors to tissue injury and fibrosis in disease (18–20, 21). However, some researchers overexpressed PRR in normal mice, no increase in blood pressure and no tissue damage were observed (22). This might indicate that PRR is not a primary initiator of tissue damage but exacerbates tissue injury in pathological conditions (22).

A truncated form of PRR named M8.9 was found to be an accessory protein of vacuolar H + -ATPase (V-ATPase) and plays a critical role in V-ATPase biogenesis (23, 24) (Figure 1D). Previous studies showed that abnormal cytoskeleton and impaired autophagy caused by the deletion of PRR significantly affected cell survival (23, 25). These studies might suggest that the deletion of PRR is more lethal than the overexpression of PRR. Although it is still controversial whether moderate knockdown of PRR under pathological conditions will lead to lysosomal dysfunction and autophagy impaired, apparently the best option would be to block PRR without affecting M8.9, and not knockdown gene expression.

Interestingly, Cruciat et al. (26) found that PRR is an important component of the Wnt receptor complex together with Frizzled, V-ATPase and a low-density lipoprotein receptor-associated protein 6 (LRP6) and participates in the activation of Wnt/β-catenin signaling, which contributes to cell development and may participate in tissue fibrosis (Figure 1C). This discovery provides another possible mechanism for the physiological and pathological effects of PRR.

### 2.2. sPRR

In addition to M8.9, in 2009, a 28-kDa soluble (pro)renin receptor (sPRR) was found in plasma (27). These findings might indicate that the full-length PRR is cleaved by some means. However, the generation of sPRR remains controversial. Cousin et al. (27) reported that PRR was cleaved by furin in the trans-Golgi, which generates a 28-kDa sPRR and an additional 10-kDa fragment. However, there is no evidence that the 10-kDa fragment is M8-9, the accessory protein of V-ATPase. In contrast, another study found that metalloproteinase ADAM19 mediates the shedding and cleavage of PRR in Golgi and generates 29 kDa NTF-PRR and CTF-PRR (28), which may suggest furin is not the only cleavage protein of full-length PRR. Recently, Nakagawa et al. (29) found that the cut site of sPRR fits well to the cleavage site of Site-1 protease (S1P), a member of the subtilisin-like proprotein convertase family. Through the use of specific S1P knockdown, they proposed that full-length PRR was first cleaved by S1P and further cleaved by furin to generate sPRR, which was secreted extracellularly (29). Furthermore, an animal experiment confirmed that inhibition of S1P effectively reduced the sPRR level in Ang II–induced hypertension mice (30), which may suggest that S1P is the rate-limiting protein in the production of sPRR. Multiple clinical studies have confirmed the relevance of sPRR and some diseases, including gestational diabetes (31), essential hypertension (32), chronic heart failure (33), and renal damage (34); however, the physiological function and pathological mechanism of sPRR have not been elucidated to date.

## 3. The roles of PRR and sPRR in cardiovascular diseases

### 3.1. Physiological state

In physiological state, PRR was discovered high expression in human brain, heart, kidney and colon (11). There are sufficient evidences suggested that PRR plays an important role in cell proliferation and cell cycle progression (35–37). This effect might be associated with Wnt/β-catenin (35). There are numerous literature reports on the essential role of Wnt/β-catenin in cell survival and proliferation, cell fate and movement (38). As part of Wnt receptor complex, PRR knockdown impairs cell proliferation and normal morphogenesis both through canonical and non-canonical Wnt/PCP signaling pathway, which leads to neurodevelopmental abnormalities in mice (35). Wanka et al. also demonstrated that PRR knockdown decreases cell proliferation and a cell cycle arrest in the G0/G1 phase in renal As4.1 cells (36). Moreover, another study showed that PRR overexpression facilitates cell proliferation in hippocampal neural stem cells (37). This finding provides further evidence of the above research.

As mentioned above, the truncated form of PRR is an important accessory protein of V-ATPase. V-ATPases are proton-pumping membrane proteins that drive protons into the lumen of lysosomes using ATP hydrolysis’ free energy (39). It contributes to maintaining the acidic environment of lysosome, and provides a conducive environment for lysosomal hydrolase activity (39). Kinouchi et al. confirmed PRR ablation decreases the expression of V0 subunits of V-ATPase and caused V-ATPase function impairment (23). Their further study demonstrated that full-length PRR participants in V-ATPase biogenesis. M8.9 might be just a residue after cleavage of full-length PRR (24). Therefore, PRR is important in maintaining normal function of lysosome in physiological state. Deletion of PRR results in lysosomal acidification disorder and impaired autophagy, and finally leads to cell death (25).

(Pro)renin receptor was first been found as a new member of RAS. Existing studies demonstrate that PRR is involved in local RAS both through Ang II-dependent and -independent ways. However, whether PRR can mediate tissue inflammation and fibrosis by local RAS under physiological conditions remains controversial. Moilanen et al. overexpressed PRR by adenovirus in normal rat hearts and observed cardiac hypertrophy, extracellular matrix fibrosis and reduced cardiac ejection fraction, accompanied by activation of the ERK1/2 and p38MAPK-HSP27 pathways (40). They also suggested that PRR stimulated the p38 MAPK-HSP27 pathway at least partially through Ang II (40). Another research found that specific overexpression of PRR in normal mice hearts caused atrial fibrillation and cardiac remodeling via ERK1/2 (41). In contrast to these studies, some studies overexpressed PRR in the heart and were unable to notice myocardial injury or cardiac fibrosis (22). They proposed that PRR may aggravate tissue damage caused by inflammation or diabetes, but not to be the primary initiator (22). Batenburg et al. demonstrated that only overexpression (pro)renin but not PRR can stimulated intracellular signaling pathway (42). They also suggested that the low (pro)renin level in normal tissue was not enough to activate PRR (42). Moilanen et al. overexpressed PRR by recombinant adenoviruses that carry rat PRR genes (40). Comparatively, Rosendahl et al. constructed PRR gene overexpression mice to up-regulate PRR expression (22). Different methods and times of PRR overexpression may be responsible for the discrepant results. But this still lacks evidence and needs further research.

Altogether, under physiological states, in contrast to the controversial results of tissue damage and fibrosis caused by PRR, its importance for maintaining cell survival cannot be disputed. Therefore, downregulation of PRR gene expression is dangerous. Clinical treatment targeting PRR should focus on its blockers.

### 3.2. Hypertension

Since the PRR was first reported (3), considerable research has revealed the association between PRR and hypertension. PRR participates in the pathogenesis of hypertension as part of local RAS rather than system RAS. Therefore, PRR is involved in hypertension through different mechanisms in different tissues.

#### 3.2.1. Brain PRR

There is continued debate about the effect of the renin–angiotensin system in the brain because the lower expression level of renin in the brain might not be sufficient to generate and activate Ang II (43, 44). The discovery of PRR provided novel insight into the controversy.

Shan et al. (45) noticed that PRR silencing in supraoptic nucleus (SON) improved blood pressure in spontaneously hypertensive rats, and overexpression of PRR in SON stimulated vasopressin (AVP) secretion in normotensive rats but did not influence blood pressure. Furthermore, they coincubated PRR and AGT, which verified PRR’s ability to facilitate Ang II generation (45). Soon after, another study showed that knockdown of PRR in the brain decreased blood pressure and reduced angiotensin II type 1 receptor (AT1) and AVP levels in Ang II–dependent hypertensive mice (4, 46). In salt-sensitive hypertensive mice, neuron-specific PRR knockout prevented the generation of Ang II in the brain (47). These studies demonstrated that PRR may play an important role in the regulation of the brain RAS system and water balance through AVP. This was corroborated by later studies in humans (48). However, these studies all denied that PRR overexpression increased blood pressure (BP) and heart rate (HR) under physiological conditions (4, 22, 45). This contradiction might be partially due to limited (pro)renin secretion resulting in lower PRR activity and insufficient activation of intracellular signaling pathways under physiological conditions, so overexpression cannot significantly increase PRR activity. Its weak BP elevating effect might be compensated by other BP regulation mechanisms. Intracerebroventricular infusion of (pro)renin in normotensive mice could increase BP (47). This result provided supporting evidence for the viewpoint. Interestingly, Villar-Cheda et al. showed that an Ang II (100 nM) treatment of MES 23.5 dopaminergic neurons increase the mRNA expression levels of PRR (49). This effect can be reversed by losartan (49). Ang II combined with AT1R can increase the expression of PRR, which may partly explain why PRR plays an important role in Ang II–dependent hypertension.

Except for the RAS, the autonomic nervous system is more integral to blood pressure regulation. Central sympathetic activity plays a key role in raising BP (50). Neuroinflammation and oxidative stress in the rostral ventrolateral medulla contribute to increased BP and mediate spontaneous hypertension (51–54). PRR has been found to be expressed in multiple cardiovascular-relevant brain regions (19, 55), including the solitary tract (NTS), supraoptic nucleus (SON), and hypothalamic paraventricular nucleus (PVN). Therefore, most subsequent studies focused on exploring the mechanism of PRR, which mediates hypertension through neuroinflammation and oxidative stress in nerve cells, independent of Ang II. Peng et al. (14) developed adeno-associated viral coding for human PRR to transfect neuronal cells *in vitro* and found that AAV-PRR increased NADPH oxidase (NOX) 2 and NOX4 mRNA levels via the MAPK/ERK1/2 intracellular signaling pathway independent of Ang II, which further stimulated reactive oxygen species (ROS) production and accumulation. They subsequently constructed a neuro-specific hPRR (Syn-hPRR) overexpression mouse model to demonstrate *in vivo* that PRR elevated BP via the ERK-NOX mechanism (56). Huber et al. (55) also verified this result and suggested that ROS accumulation in the PVN mediated by PRR stimulated sympathetic activation, which further caused increased arterialBP. Moreover, Hu et al. (57) found that the blood pressure-raising effect may be associated with the NOD-like receptor family pyrin domain containing (NLRP) 3 inflammasomes. These are multiprotein complexes that lead to the release of the proinflammatory cytokines interleukin 1 beta (IL-1β) and IL-18 (58). NLRP3 can be activated by a variety of stimuli (59, 60), including the accumulation of ROS and damaged mitochondria (61, 62). Based on this, Hu et al. (57) elucidated that ROS accumulation caused by PRR triggers NLRP3 activation and facilitates M1 proinflammatory phenotype switching of microglia. These studies revealed the BP regulatory effect of PRR through neuroinflammation and oxidative stress in the brain, independent of Ang II. This is another mechanism of PRR-mediated BP regulation in the brain and may provide a new direction for the treatment of neurological hypertension.

#### 3.2.2. Kidney PRR

It is well known that the kidney is a crucial organ in blood pressure regulation and fluid balance. Studies have affirmed that PRR promotes local renin–angiotensin system activation in the renal medulla and collecting duct (63–66). However, the mechanisms in the kidney might be different from those in the brain.

Cyclooxygenase (COX) 2, as a rate-limiting enzyme in the conversion of arachidonic acid to prostaglandins, is a classic and important therapeutic target in the clinic (67). It also shows a certain effect in increasing renin activity in the kidney (68). Soon after the discovery of PRR, research reported that the mRNA expression of COX2 in the renal cortex increased in human PRR gene-transgenic rats (69). This result suggested that there is a modest association between PRR and COX2 expression. Gonzalez et al. (70) demonstrated that PRR facilitated COX2 expression independently of Ang II in rat renal inner medullary cells via ERK1/2 activation. Their further investigation found that PRR and COX2 expression levels were both increased in the renal medulla and contributed to blood pressure elevation in Ang II-dependent hypertensive mice. A COX2 inhibitor partially rescued blood pressure at 14 days (71). This finding suggested that COX2 expression mediated by PRR may contribute to Ang II-dependent hypertension. Interestingly, some researchers suggested that COX2 elevated PRR expression through the COX2-derived product prostaglandin (PG) E2 in the renal medulla of Ang II-dependent hypertensive mice (72). Then, they illustrated that the prostaglandin e-prostanoid 4 (EP4) receptor might be a crucial contributor to this process as a PGE2-specific receptor (73). These results indicated that there may be positive feedback between PRR and COX2 (74), and it played an important role in Ang II-dependent hypertension. Furthermore, aquaporin 2 (AQP2), which mediates urine concentration and water retention, might be a downstream target of PRR activated by PGE2-EP4, and this sequence is induced by AVP (75). As mentioned earlier, AVP can be upregulated by PRR in the brain, which indicates that there may be more complicated feedback of PRR in blood pressure regulation.

The sodium reabsorption function of renal tubules plays a significant role in vascular volume homeostasis and blood pressure regulation. PRR has been found to be expressed in the proximal tubules (PT), medullary thick ascending limbs (MTAL), and collecting ducts (CD) (63, 76). Peng et al. (77) first reported that PRR induced the expression and activation of renal medullary α-epithelial sodium channels (α-ENaC) in Ang II-dependent hypertensive rats. A similar result that PRR increased blood pressure via α-ENaC was confirmed in obesity-induced hypertension rats (78). The study also suggested that the activation of α-ENaC might occur through the activation of serum/glucocorticoid-regulated kinase 1 (SGK-1), the primary regulator of α-ENaC, via the PI3K-AKT pathway (78). Shortly after that, Xu et al. (66) corroborated that high fructose increased PRR expression, which stimulated sodium/hydrogen exchanger 3 and Na/K/2Cl cotransporter upregulation and caused salt-sensitive hypertension. In connection with the activation effect of AQP2, these results provide evidence for the role of PRR in the retention of water and sodium and blood pressure regulation through ion transporters in renal tubules.

However, as an essential accessory protein of V-ATPase, the absence of PRR leads to severe impairment in the function of V-ATPase (23, 24, 79, 80). Downregulation of important ion transporters caused by V-ATPase dysfunction was confirmed in nephron-specific PRR knockout mice and further resulted in renal concentration defects and distal renal tubular acidosis (79). V-ATPase dysfunction caused by PRR absence commonly occurs prenatally in PRR knockouts (23, 79), which suggests that appropriate PRR blockade in the therapy of hypertension may not lead to a serious adverse consequence in adults, but further experiments are required for validation.

#### 3.2.3. Plasma sPRR

Clinical research has revealed that in essential patients, levels of serum sPRR levels correlated positively with serum creatinine levels, but had no correlation with BP (32). In recent years, since S1P was verified as a new cleavage site of full-length PRR, the effect of S1P-driven sPRR was uncovered in hypertension model mice. Wang et al. (30, 81) demonstrated that S1P blockade improved Ang II-induced hypertension by decreasing the activation of ENaC and AQP2 in the kidney, and this effect was reversed by intravenous administration of recombinant sPRR. This finding suggested that S1P-derived sPRR might be involved in the pathogenesis of hypertension. Other researchers infused recombinant sPRR (30 μg/kg⋅day) in high fat-fed male mice and observed impaired baroreflex sensitivity and sympathetic outflow, which increased BP (82). Furthermore, to exclude the potential influence of S1P knockout and clarify the effect of S1P-derived sPRR, Ramkumar et al. (83) used CRISPR–Cas9 to specifically mutate the cleavage site of the PRR, and plasma sPRR levels were virtually undetectable, which reduced BP and decreased renal injury in Ang II-induced hypertension mice. Interestingly, another study suggested that sPRR might directly bind and activate the AT1 receptor, which caused increased blood pressure and endothelial dysfunction in obesity-related hypertensive mice (84). This finding implies that Ang II is not the only way to activate AT1R and can help to further understand the RAS system. These findings suggest the potential of sPRR in the diagnosis and therapy of hypertension, warranting further investigation.

### 3.3. Myocardial ischemia reperfusion injury

Myocardial ischemia reperfusion injury (MIRI) often occurs after myocardial infarction reperfusion therapy and causes further injury. Liu et al. (85) treated cardiomyocyte cells (H9C2) with 2h of hypoxia followed by 6 h of reoxygenation to mimic ischemic-reperfusion injury *in vitro* and found that PRR expression was upregulated. PRR small interfering RNA reduced p38-MAPK activation and decreased hypoxia/reoxygenation-induced apoptosis via p38-MAPK (85). Furthermore, some researchers demonstrated that PRR overexpression increased apoptosis and autophagy in H9C2 cells treated with hypoxia/reoxygenation condition (86). They first proposed that this effect may occur through the Wnt/β-certain pathway because a Wnt inhibitor (DKK-1, 20 ng/ml) reversed the above effects (86), though this result has not been validated *in vivo*.

### 3.4. Heart failure and cardiac remodeling

#### 3.4.1. PRR

At present, reports on the study of PRR in heart failure are scarce. In 2012, Rademaker et al. (87) evaluated PRR blockade handle region peptide (HRP) (1, 5, and 25 mg) in sheep with heart failure and found that PRR blockade decreased atrial pressure and Ang II levels and improved renal function. Another study showed that PRR blockade (HRP, 0.3 mg/kg) attenuated fibrosis and hypertrophy by decreasing ERK1/2 activation and TGF-β expression in mice, ameliorating heart failure caused by chronic kidney disease, with no effect on autophagy (88). Moreover, the latest literature reports that PRR blockade reduced ROS generation and endoplasmic reticulum stress and increased cAMP levels. These biological processes attenuate cardiac remodeling in heart failure (89). These studies are consistent in that PRR blockade improved systolic blood pressure and reduced ventricular preload in chronic heart failure mice. The mechanism of the antihypertensive effect of PRR blockade and knockdown in heart failure is still not clear but might be associated with the lower level of local Ang II production. Moreover, PRR blockade has no effect on autophagy, which means that its effects on lysosomal function and cell survival should not be contraindicative. These findings are encouraging for the possible clinical application of PRR blockade.

However, our recent study indicated that in mice with heart failure induced by transverse aortic constriction (TAC) surgery, adenovirus-mediated gene silencing of PRR leads to autophagic flux blockade, which causes an imbalance in ROS production and scavenging and eventually results in cardiac dysfunction and fibrosis (90). M8.9 is a crucial part of V-ATPase. Autophagy impairment still cannot be avoided in the knockdown of PRR genes. However, the therapeutic effects of PRR blockade in heart failure mice cannot be ignored. These studies suggest that we should search for ways to block PRR but not to decrease the gene expression of PRR.

#### 3.4.2. sPRR

At present, few studies have focused on the effect of sPRR in heart diseases, and only a small number of clinical studies have shown high sPRR levels in heart failure patients (33). However, recent studies on sPRR, especially the effect of sPRR activating AT1R, implied that sPRR might also participate in heart disease pathogenesis, which requires further study. Some researchers have suggested that sPRR led to tissue fibrosis via the PI3K-AKT pathway and could also cause oxidative stress via NOX4 in renal proximal tubular cell lines (91, 92). It is still not clear whether sPRR has the same effect on the myocardium. This issue can be investigated in the future. However, it should be considered whether the therapeutic effect of full-length PRR knockdown on heart disease was due to reduced sPRR levels. Some studies did not support this conjecture because the application of an AT1R inhibitor (losartan) does not reverse the myocardial remodeling caused by PRR overexpression. However, we still need to pay attention to this issue in future research; that is, investigation of full-length PRR needs to exclude the effects of sPRR.

On the other hand, a clinical study found that there is an association between high plasma sPRR levels and left ventricular remodeling, particularly renal dysfunction in chronic heart failure patients with reduced ejection fraction (93). Another clinical study supported this conclusion. It showed that higher plasma concentrations of sPRR were associated with lower left ventricular ejection fractions and greater degrees of dilatation in the left ventricle in elder chronic heart failure patients (33). These studies revealed the potential of sPRR as a biomarker of chronic heart failure. The sPRR might be applicable to assessing the severity of chronic heart failure in clinical practice, but further research is necessary.

### 3.5. Metabolic cardiomyopathy

**FIGURE 2:**
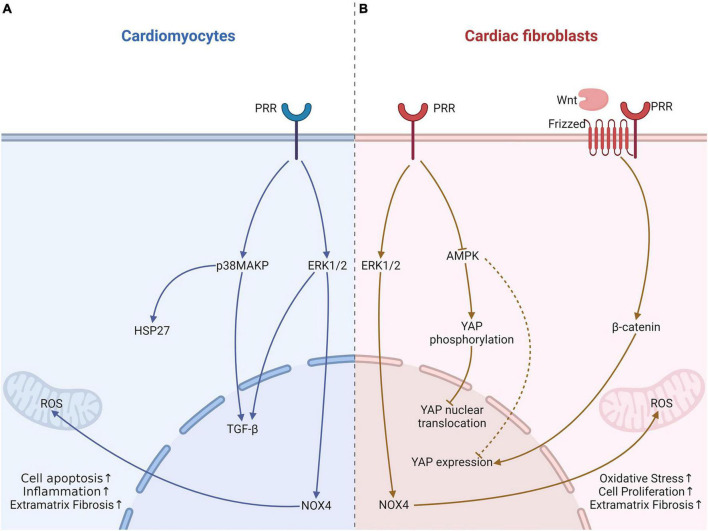
The effects of PRR on cardiomyocytes and fibroblast in heart tissue with metabolic cardiomyopathy. **(A)** In cardiomyocytes, PRR promotes cardiomyocytes apoptosis via p38MAPK-HSP27, facilitates TGF-β expression via ERK1/2 leads to fibrosis, and causes oxidative stress through increasing NOX4 expression. **(B)** PRR promotes YAP expression, further causes fibroblast proliferation and myocardial fibrosis. This effect of PRR maybe associate with AMPK and Wnt. PRR may also regulates YAP phosphorylation and nuclear translocation.

Metabolic cardiomyopathy is a type of secondary cardiomyopathy, often secondary to basal metabolic diseases, including diabetes, obesity and alcohol intake (94). Diabetes is the most common disease of these patients. Our previous study demonstrated that PRR RNA interference silencing attenuated the inflammatory response, cardiomyocyte apoptosis and myocardial fibrosis in (DCM) (5, 95), which can be associated with lower NOX4 expression (95). NADPH oxidase (NOX4) not only increases ROS production but also mediates fibroblast proliferation and might further activate ERK1/2 and p38 MAPK (96), which may be an important mechanism by which PRR causes cardiac fibrosis. We also showed that PRR upregulated inflammatory factor expression, induced cardiomyocyte apoptosis and myocardial fibrosis, and promoted ROS production (5). These effects can be reversed by an ERK inhibitor in fibroblasts stimulated by high glucose (5). In addition to DCM, our study also showed that a similar effects of PRR in alcoholic cardiomyopathy model rat, which was that PRR promoted myocardial fibrosis in alcoholic cardiomyopathy (ACM) rats via PRR-ERK-NOX4 (6) (Figure 2). These *in vivo* and *in vitro* studies imply that PRR might be a new therapeutic target in metabolic cardiomyopathy.

Adenosine 5′-monophosphate-activated protein kinase (AMPK) is a crucial enzyme in regulating energy metabolism homeostasis and stress, and it contributes to pathogenesis of type 2 diabetes (97). Recent research suggested that in diabetes, PRR can decrease AMPK phosphorylation, which might mediate mitochondrial biogenesis and result in function impairment (98). Our previous study verified that PRR reduced AMPK phosphorylation in DCM (99). Our recent research also found that PRR mediated cardiac inflammation and fibrosis in DCM, at least partly through enhancing yes-associated protein (YAP) expression (100). YAP is a key protein that mediates mechanical signaling and cell proliferation (101) and promotes the transcription of downstream target genes through nuclear translocation. Phosphorylation of YAP causes its cytoplasmic localization, which prevents YAP nuclear translocation and further decreases target gene activation (101). Recent studies found that YAP promoted fibroblast differentiation and activation and increased extracellular matrix fibrosis (102). Tissue fibrosis was attenuated by selective knockout of YAP expression (102). Our studies illustrated PRR overexpression increased YAP expression, and a YAP inhibitor could reverse PRR-mediated cardiac fibrosis (99). Furthermore, the PRR-induced increase in YAP expression might occur partly through the downregulation of AMPK phosphorylation (99, 100). In our experiments, we have not clarified whether PRR regulates YAP phosphorylation and nuclear translocation. However, studies have elucidated that AMPK promotes YAP phosphorylation and prevents its nuclear translocation (103). PRR may also increases YAP nuclear translocation through down-regulation of AMPK in DCM. Moreover, Yoshida et al. (104) showed the association of PRR and YAP, presumably through the activation of the Wnt/β-certain pathway. YAP was regarded as a downstream effector of Wnt/β-certain (105) (Figure 2). Further research is needed to find out the relationship among PRR, Wnt/β-certain and YAP pathway, and their roles in cardiac remodeling.

### 3.6. Lipid metabolism and atherosclerosis

Atherosclerosis is the most common vascular disease characterized by arterial wall thickening and atherosclerotic plaque or lesion formation and results in coronary heart disease (CAD), cerebral infarction, myocardial infarction and multiple diseases (106). In the pathogenesis of atherosclerosis, lipid metabolism disorder is the pathological basis of atherosclerosis. Lipid deposition and lipid-phagocytosed macrophage accumulation play vital roles in the formation of arterial plaques (107, 108). There is compelling clinical evidence that dyslipidemia is significantly associated with the risk of atherosclerosis. In particular, low-density lipoprotein (LDL), as the cholesterol carrier protein, is an independent factor for predicting the risk of cardiovascular disease (108, 109). Interestingly, the present study found that PRR was a new component of the lipid metabolism pathway (110), which suggests that PRR might participate in atherosclerosis in this manner (111).

Lu et al. (110) confirmed sortilin (SORT) 1, a recently identified key regulatory protein of LDL metabolism, as a PRR-interacting partner through an unbiased proteomics approach. They demonstrated that PRR gene silencing decreased SORT 1 abundance but without significantly reducing the mRNA expression of SORT 1, and PRR gene silencing also decreased LDL receptor abundance, which impaired LDL uptake (110). These effects might be related to PRR depletion mediating V-ATPase dysfunction and lysosome impairment because the LDLR abundance reduction could be partly rescued by lysosomotropic agents (110). Strong et al. (111) suggested that PRR advances SORT1 and LDLR transport to the cell surface, and SORT1 protects LDLR from degeneration by lysosomes. This hypothesis provides a possible model for the interaction of PRR, SORT1 and LDLR. However, unexpectedly, in the current research (112), PRR gene silencing even reduced plasma low-density lipoprotein cholesterol (LDL-C) and triglycerides. They elucidated that this effect might be due to PRR inhibition reducing the abundance of acetyl-CoA carboxylase (ACC) and pyruvate dehydrogenase (PDH) in hepatocytes, resulting in reduced lipid synthesis (112). As a consequence, hepatic PRR inhibition improved diet-induced obesity and liver steatosis (112). Interestingly, Gatineau et al. (113) showed that liver PRR knockout might compensate for elevated sPRR cleavage and secretion from adipose tissue, which increased sterol regulatory element-binding protein 2 (SREBP2) expression and hepatic cholesterol synthesis. Taken together, hepatic PRR and sPRR both contribute to lipid synthesis and LDL uptake in intricate ways, which affect plasma LDL levels and may cause atherosclerosis and CAD.

In addition to lipid synthesis and metabolism, migration and proliferation of vascular smooth muscle cells (VSMCs) are also key events in atherosclerosis and vascular remodeling. Previous studies have shown that (pro)renin combines with PRR in smooth muscle cells, which contributes to the migration and proliferation of VSMCs by regulating plasminogen activator inhibitor-1 (PAI-1) expression (114, 115). Further studies confirmed (pro)renin as a potential chemotactic factor. When combined with PRR, it activates RhoA-GTP and Rac1-GTP and promotes the cleavage of focal adhesion kinase (pp125FAK), which causes cytoskeleton reorganization and VSMC migration (116). Some studies questioned the significance of these conclusions because the (pro)renin level of vascular smooth muscle did not reach the concentration that activated PRR (42). However, in our recent study, we still found that overexpressed PRR facilitated the formation of Ang II-induced abdominal aortic aneurysm in apolipoprotein E-knockout mice (7). These conflicting conclusions might be because PRR is not only activated by (pro)renin or renin but can also be directly activated by Ang II or others. This conjecture has received indirect support from some research, but it still needs further verification.

## 4. The inhibitors of PRR

Soon after PRR was identified, to inhibit its adverse effect, Ichihara et al. (12) synthesized a 10 aa length bait peptide, called handle-region peptide (HRP), which could competitively combine with PRR and was expected to be used for clinical treatment by blocking the combination of (pro)renin and PRR. The applications of HRP have demonstrated some degree of renal and cardiovascular protective effects in multiple disease models, including experimental heart failure (117), ameliorated heart failure (88), type 1 diabetes and diabetic nephropathy (118, 119). However, the effectiveness of HRP is controversial. Many researchers have questioned the effects of HRP on PRR blockade and the treatment of hypertension and diabetes. Feldt et al. (120) suggested that HRP bound to the U937 cell surface, but the ERK1/2 activation induced by (pro)renin and renin was not inhibited by HRP. They also found that HRP treatment had no effect on renal injury or blood pressure in hypertensive nephrosclerosis rats (121). The combined treatment of HRP with aliskiren, a renin inhibitor, had no additive protective effects on target organs in diabetic nephropathy rats (122). Indeed, it even offsets the protective effect of aliskiren in combination medication (123, 124). In conclusion, although in some animal models, HRP plays a possible protective role in renal and cardiac remodeling, it is apparently disappointing as a PRR inhibitor in the treatment of diseases.

To further explore the method of blocking PRR and applying it to treatment, Li et al. (125) synthesized a novel antagonistic peptide, named PRO20, which is the first 20 amino acids of the (pro)renin prosegment, and interestingly, it combined with PRR and successfully inhibited Ca2 + influx and ERK1/2 activation when intraventricularly injected in rats with deoxycorticosterone acetate-salt–induced hypertension (125). Other studies infused PRO20 into the renal medulla and found that it reduced sodium–water retention and attenuated Ang II-induced hypertension (77). It has also been reported to improve nephrectomy-induced nephropathy in rats by inhibiting Wnt/β-catenin signaling (126). It is exciting that PRO20 can inhibit Wnt/β-catenin signaling. Another study showed that PRO20 also increased intracellular cAMP levels and reduced endoplasmic reticulum (ER) stress in cardiac remodeling (89). This finding suggested that PRO20 may not only be used for hypertension but also has some potential in the treatment of cardiac remodeling, but further research is needed.

## 5. Conclusion

In this review, we summarized and discussed the roles of PRR and its soluble form in cardiovascular diseases. At present, as a new therapeutic target, full-length PRR is still quite controversial. Further research is needed on PRR inhibitor and its physiological and pathological effects. Moreover, recent studies have focused on sPRR and achieved significant advancements. Whether it can be used as a prognostic indicator and therapeutic target for cardiovascular disease may be a potential direction for further research.

## Author contributions

BW wrote this review and drew the figures. HJ assisted in reviewing literature and writing the manuscript. BD, SW, and YZ conceived and guided the writing. All authors contributed to the article and approved the submitted version.
